# The association between sleep spindles and cognitive performance in euthymic bipolar disorder

**DOI:** 10.1038/s41398-026-04273-2

**Published:** 2026-07-14

**Authors:** Anna Tröger, Jules Schneider, Dimosthenis Tsapekos, Stephan Bialonski, Niklas Grieger, Michail Kalfas, Lisa Frending, Hannah Hartland, Heidi Kuivaniemi-Smith, Allan H. Young, Philipp Ritter, Rebecca Strawbridge

**Affiliations:** 1 Department of Psychological Medicine, Institute of Psychiatry, Psychology & Neuroscience, King’s College London, London, UK; 2 Department of Psychiatry and Psychotherapy, University Hospital Carl Gustav Carus, Technische Universität Dresden, Dresden, Germany; 3 Department of Medical Engineering and Technomathematics, Institute for Data-Driven Technologies, FH Aachen University of Applied Sciences, Jülich, Germany; 4 Department of Information and Computing Sciences, Utrecht University, Utrecht, The Netherlands; 5 Department of Brain Sciences, Faculty of Medicine, Imperial College London, London, UK; 6 Department of Psychiatry, University of Oxford, Oxford, UK

**Keywords:** Diagnostic markers, Learning and memory, Human behaviour, Bipolar disorder

## Abstract

Many people with bipolar disorder (BD) experience persistent cognitive deficits. Sleep spindles have been linked to cognitive ability in healthy populations and psychotic disorders. While there is preliminary evidence for altered spindle activity in BD, research directly examining the association between sleep spindle parameters and cognitive performance in this population is lacking. Therefore, our primary objective was to examine the association between fast spindle density and episodic memory performance in euthymic individuals with BD. As exploratory analyses, we looked at associations between fast and slow spindle density and subjective sleep quality with other cognitive domains. We also conducted a sensitivity analysis, separating all analyses by lithium intake. Thirty-four euthymic participants with BD underwent comprehensive cognitive assessments and were assessed for three consecutive nights using mobile sleep-EEG headbands. Sleep spindles were detected using validated, adapted algorithms and characterised by fast and slow spindle density. Fast spindle density was not associated with episodic memory performance (*r* = −0.004, *p* = 0.491, 95% CI [−0.353, 0.499]) but did show a significant positive association with working memory (*r* = 0.423, *p* = 0.014, 95% CI (bootstrapped) [−0.167, 0.722]). When removing participants taking lithium, several positive associations emerged between fast spindle density and cognitive performance. These findings suggest domain-specific relationships between sleep spindle activity and cognition in BD, with fast spindles potentially being associated with working memory. Preliminary evidence for lithium-related modulation highlights the importance of considering pharmacological factors. However, the analyses were underpowered, and large-scale studies are needed to deepen our understanding of sleep spindle-cognition relationships in BD.

## Introduction

Bipolar disorder (BD) is a chronic affective disorder that affects more than 1% of the global population and constitutes one of the main causes of disability [1]. Many people with bipolar disorder experience persisting cognitive difficulties, such as impairments in memory, attention, or executive functioning [2, 3]. These difficulties can persist even in euthymia [4], may negatively impact their quality of life and psychosocial functioning, and can pose a risk for affective relapse [5, 6]. Recent studies suggest a heterogeneous cognitive profile between individuals, ranging from relatively intact to severely impaired cognition and longitudinal trajectories that suggest premorbid cognitive difficulties for some and cognitive decline only after illness onset for others [7, 8]. Premorbid IQ is clearly relevant to consider when characterising cognitive impairment, with normative approaches potentially underestimating substantially the extent of deficits for many individuals [9]. To develop and optimise psychological and pharmacological intervention to combat these cognitive impairments, it is important to understand their underlying biological and neural mechanisms. This can be critical for early detection and nosological clarification, especially in distinction to similarly presenting mental disorders, such as major depression or schizophrenia. Electroencephalography (EEG) can be a useful tool in this endeavour, as it is non-invasive, and directly measures brain activity with high temporal resolution [10]. A recent systematic review on wake-EEG markers of cognitive performance in BD from members of our group examined direct correlations between different EEG measures (e.g., ERP measures, spectral measures) and cognitive performance in various cognitive domains but did not identify a robust marker of cognition within wake-EEG measures [11].

A promising alternative could be the identification of mechanisms related to cognition during sleep through sleep-EEG. Sleep disturbances have consistently been linked to poorer cognitive performance, steeper cognitive decline as well as impaired memory formation and consolidation in healthy populations [12, 13], and sleep abnormalities as well as poor sleep quality are common in BD across mood phases [14, 15]. Indeed, a recent systematic review found preliminary evidence for an association between sleep disturbances and cognitive impairments in affective disorders [16]. A specific parameter that may play a crucial role in this interaction is sleep spindles. Sleep spindles are brief (approximately 0.5–3 s) bursts of oscillatory activity within an 11–16 Hz frequency range that occur in adult NREM sleep, predominantly during stage 2 (N2). They are commonly distinguished into more frontal, slow (≤13 Hz) and typically centroparietal, fast (>13 Hz) spindles [17]; however, other cut-offs (e.g., 14 Hz; [18]) and methods adjusting for individual spindle frequency differences [19] have also been reported in the literature. Sleep spindles are generated through thalamo-cortico-thalamic feedback loops and provide neural conditions favourable for sensory gating (thus protecting NREM sleep from disruption), interareal cortical communication, as well as learning, memory consolidation and activation [17, 20]. There is solid evidence for the association of sleep spindle measures with cognitive ability/IQ in healthy adults [21]. Additionally, neurobiological models that plausibly link hippocampal ripples to cortical slow oscillations via sleep spindles, thus facilitating the stable encoding of new memories, exist [17]. In psychotic disorders, including schizophrenia, (fast) sleep spindle measures, specifically density, are often reduced compared to healthy control subjects [22, 23]. Moreover, there is strong evidence for associations between sleep spindle density and different cognitive parameters in psychotic disorder patients and their first-degree relatives [[19, 24–27]; but see [28] for no significant associations], usually suggesting reduced spindle density to be associated with reduced cognitive performance. A meta-analysis by Au and Harvey [18] on spindle activity and cognitive functions in psychosis confirmed an increase in sleep spindle activity being correlated with improvement in at least one cognitive domain, with moderate-to-strong associations; processing speed and attention were the most consistently correlated cognitive domains, while spindle density was the most consistent spindle measure. Notably, some research also suggests excessive spindle density to be associated with some neurodevelopmental disorders [17].

For BD, the overall evidence is scarcer and less conclusive. A review of EEG sleep abnormalities in BD [29] only identified two papers reporting on sleep spindles in this population [30, 31], which both found a significantly lower (fast) spindle density in BD compared to controls. However, preliminary results from a high-density EEG study showed no significant differences in any sleep spindle parameter [32]. Moreover, lithium, a medication primarily prescribed for BD, may modulate sleep and cognition in BD. For example, previous research has preliminarily suggested pro-cognitive effects of lithium in BD [33] and has highlighted its potential impact on the circadian clock [34]. To the best of our knowledge, no study has yet investigated associations between sleep spindles and cognitive performance in BD, including the consideration of lithium use as a putative moderating factor.

Recent advances in sleep-EEG technology, like wearable EEG headbands, have the potential to facilitate sleep research on a larger scale. Further, automated sleep stage scoring and spindle detection can match expert-level performance, while being significantly faster than a human scorer and without intra-rater variability [17, 35].

Given the potential of sleep spindles as a mechanism of cognition in BD and a current lack of such an investigation, we aimed to analyse the association between sleep spindle density and cognitive performance (memory performance, global cognition composite score, and individual test scores), as well as subjective sleep quality, using wearable EEG headbands in euthymic BD patients. Our primary objective was to examine the association between fast spindle density and episodic memory performance, hypothesizing a positive correlation between the two. As exploratory analyses, we looked at associations between fast and slow spindle density and subjective sleep quality with other cognitive domains. We also conducted a sensitivity analysis, separating all analyses by lithium use.

## Materials & methods

### Design

The present study is an analysis of baseline data from a subset of participants from the multisite randomized controlled trial (RCT), Cognitive Remediation in Bipolar Disorder 2 (CRiB2; for details see [36]). CRiB2 assessed the effectiveness of cognitive remediation therapy (CRT) compared to treatment as usual in improving cognitive and psychosocial functioning in BD. It was reviewed and approved by the London – Bromley research ethics committee (REC) in Spring 2022 (22/LO/0210) and was conducted in accordance with the declaration of Helsinki [37]. The study recruited participants from July 2022 until July 2025, with the EEG component commencing in December 2023. All participants gave informed consent prior to participation. The preregistration of the present analysis can be retrieved under https://doi.org/10.17605/OSF.IO/3NKE2.

Participants were recruited through secondary/tertiary care services within the local NHS trust of each site (for the present study: South London and Maudsley (SlaM) and Oxford Health), primary care services (i.e., GP practices) in collaboration with the National Institute for Health and Care Research (NIHR) Clinical Research Network, and the community through online/in-person advertising and in collaboration with mental health charities (e.g., Bipolar UK). Participants were also identified through electronic medical records, if they had previously consented to be contacted for participation in research projects.

### Participants

To be included into the CRiB2 trial, participants needed to be: 1) diagnosed with BD-I or BD-II according to DSM-5 criteria, validated with the Mini International Neuropsychiatric Interview (MINI) 7 [38], 2) euthymic according to the Newcastle Euthymia Protocol, requiring a score of less than 8 on both the Hamilton Rating Scale for Depression (HAMD; [39]) and Young Mania Rating Scale (YMRS; [40]) covering the entire month prior to inclusion, 3) not undergoing medication changes, 4) between 18 and 65 years old, have 5) no comorbid alcohol/substance use diagnosis in the past 12 months (assessed using the MINI 7), and 6) no indications of age-related cognitive decline (assessed using the Montreal Cognitive Assessment – Telephone version [MoCA-T]; [41]) or impairing organic neurological disorder (assessed using patient-report and consultation with a medical practitioner; see [36] for full in- and exclusion criteria).

Participants were not eligible for the sleep component, if they 1) had a diagnosed or probable sleeping disorder, such as sleep apnoea, narcolepsy or REM-sleep-behaviour disorder (assessed using patient-report and the STOP-BANG questionnaire for sleep apnoea [42], on which they had to score as ‘high risk’ to be excluded), 2) had a disorder associated with significantly impaired sleep, such as polyneuropathy or chronic back pain (assessed using patient-report), 3) had an inflammatory condition, a neurological condition, or any other condition likely to interfere with EEG interpretation, such as epilepsy, cerebral palsy, or multiple sclerosis (assessed using patient-report), 4) currently used melatonin, benzodiazepines, zopiclone, or zolpidem on a regular basis, 5) had recent (within past two weeks) trans meridian travel (across three time zones), or 6) currently performed shift- or night-time work.

### Sample size rationale

The sample size was based on previous studies examining the association between sleep spindle activity and cognitive performance in psychosis that found correlations from *r* = 0.45 to *r* = 0.75 [18]. For participants with bipolar disorder, we conservatively estimated an effect of *r* = 0.45. With α = 0.05, power = 0.90, and a one-tailed test, this resulted in a required sample size of *n* = 36.

### Procedures and sleep EEG assessments

After a short initial phone call to discuss the study content and broad eligibility criteria, interested participants were screened in a one-hour phone call two weeks before the baseline assessment (screening A) and in a five- to ten-minute phone call one day before the baseline assessment to check whether their mood remained stable (screening B). Additionally, participants were asked whether they wanted to participate in the optional sleep component, which comprises the focus of the present study. If they expressed interest, they were given more information and were screened for eligibility. Eligible participants were invited to the clinical research facility at the London or Oxford site. Assessments were conducted in a noise-reduced room equipped with a table and chairs and lasted between three to four hours. At baseline sociodemographic (e.g., age, gender, ethnicity), clinical (e.g., BD-diagnosis) and service use information (e.g., past/current pharmacological and non-pharmacological treatments) was collected and the cognitive assessments were conducted.

The sleep EEG assessment was obtained utilising a mobile EEG device (Zmax by Hypnodyne Corp., Sofia, Bulgaria) at the participants’ homes. Zmax is a mobile EEG headband comprised of an amplifier/transmitter and a wet hydrogel electrode strip containing two electrodes equivalent to F7 and F8 as well as a ground and a reference electrode both equivalent to FPz with a sampling frequency of 256 Hz. During recordings, sleep EEG data was locally stored on SD cards. Zmax has been validated as a reliable device for mobile sleep EEG assessments [43]. During the baseline assessment, participants received the EEG headband, the reusable electrode strip, a charger and an instruction sheet. Participants were instructed to practice mounting and activating the device preferably in front of a mirror under supervision of study staff. They then used the device at home for three, preferably consecutive, nights within one week after the baseline visit before returning it to the study team. Three nights were chosen in order to balance data quantity and participant comfort, as three nights were thought to provide sufficiently high-quality data while keeping the potential impact on the participants’ sleep and routines at a minimum. To support adherence, participants received access to an instructional video and were encouraged to contact study staff via email or telephone with any technical difficulties during data collection. Upon device return, all recordings were transferred to the study database using Hypnodyne software (HD Format and HD Recorder) and subsequently erased from the SD card. Access to sleep recordings was limited to staff directly involved in the sleep protocol.

Participants were monetarily reimbursed for both their participation in the CRiB2 and in the sleep component study according to local standards. They also received a picture of their sleep stages for each recorded night.

### Cognitive measures

The primary outcome for the current study was an episodic memory composite score, comprising the verbal paired associates I (VPA1; [44]) and the verbal paired associates II tests (VPA2: [44]; see statistical analysis). For the VPA1, participants are given four trials to learn a set of related and unrelated word pairs. Participants hear a list of fourteen word pairs read aloud. After each learning trial, they are presented with the first word of each pair and asked to recall the associated word. The full list is then repeated in a randomised order, and this procedure is repeated across trials. The VPA2 is administered after a delay period with the same list of fourteen word pairs and without any intervening learning trials. Both measures are recognized as robust measures of memory, with good reliability and validity [45–47].

Other tasks to assess cognitive performance included the digit span test [48] to assess working memory, the trail making A test (TMT-A; [49]) to assess attention, the digit-symbol coding test [47] to assess processing speed and the FAS letter fluency test [50], the Hotel Test [51] and the Trail Making B test (TMT-B; [49]) to measure executive functions. Premorbid IQ was estimated using the Test of Premorbid Functioning (TOPF; [52]). Moreover, a global cognition composite score was calculated from all cognitive domain scores (see statistical analysis). All tasks were completed using standard templates in pencil and paper format (see [36] for a full list of measures). Subjective sleep quality was measured with the Pittsburgh Sleep Quality Index (PSQI; [53]), with higher scores indicating worse sleep quality.

### EEG data preprocessing and quality control

Each recording was band-pass filtered (0.3–35 Hz) using MATLAB for visual inspection. For the quality control check, one author (AVT) visually inspected all epochs of each recording using the SchlafEin [54] script in MATLAB, and marked all epochs in which at least 50% of data was not scorable similarly to criteria described by Esfahani et al. [43]: Epochs were excluded, if 50% of data 1) was noisy, characterised by high power across frequency ranges, 2) was lost, indicated by the absence of power throughout the frequency range or 3) contained technical glitches, indicated by unnatural waveforms. If more than 40% of epochs of one recording were excluded, the whole recording was discarded. Recordings were also discarded when they were very short (i.e., less than 5 h, unless the participant confirmed that this was all the sleep they had that night) or when the participant had informed us that they had taken or switched off the headband during the night, so the recording did not represent a whole night of sleep. All doubts about in- and exclusion of epochs and recordings were discussed with a second author (JS).

### Spindle analysis

Sleep stages and spindles were automatically detected using an adapted version of the validated algorithms in SomnoBot.org (version 1.0.0). For sleep staging, EEG signals were extracted from both channels (F7 and F8). The signals were band-pass filtered between 0.2 and 30 Hz, resampled to 60 Hz, and normalized by subtracting the median and dividing by the interquartile range. Amplitudes below −20 or above 20 were clipped to minimize outliers. Finally, sleep stages were detected using a validated neural network (RobustSleepNet; [55]) based on these pre-processed channel data.

For spindle detection, signals from each channel were used separately. The data was divided into continuous N2 sleep segments using the sleep stages detected by the RobustSleepNet algorithm, which were then band-pass filtered between 0.3 and 30 Hz and resampled to 100 Hz. Similar to the sleep staging process, each data segment was normalized by subtracting the median and dividing by the interquartile range, and amplitudes below −20 or above 20 were clipped. Spindle detection was performed using the validated SUMOv2 algorithm [35]. For the purpose of this study, SUMOv2 was fine-tuned using eight expert-annotated (JS and AVT), 30-minute EEG segments of N2 sleep recorded with a Zmax device to adapt the model to the mobile recording setup of pre-frontal channels. The detected spindles were post-processed by merging spindles with a duration of less than 0.3 seconds and a distance of less than 0.1 seconds and filtering out spindles with a duration of less than 0.3 seconds or more than 2.5 seconds [56]. A fixed cut-off to distinguish between slow and fast spindles at 13 Hz was used in accordance with Ritter et al. [31] examining sleep spindles in BD.

Spindle metrics were calculated on the raw data and determined for all, slow, and fast spindles separately. Spindle duration was measured as the time between on-and offset of each spindle. To calculate the frequency of a spindle, first, a 4^th^-order Butterworth band-pass filter (9–16 Hz) to the unprocessed EEG signal was applied. Then, spindle frequency was calculated as the average of the instantaneous frequencies, which were determined as half the reciprocals of the zero-crossing intervals of the band-pass filtered signal. Spindle amplitude was determined by subtracting the minimum amplitude from the maximum amplitude of the unfiltered signal. Afterwards, spindle outliers were excluded separately for each channel: For spindle duration, frequency and amplitude, values above or below three times the interquartile range (added to the first and third quartiles, respectively) were classified as outliers. Spindles that contained an outlier in one or more of these parameters were excluded from the dataset. Then, spindle density scores were calculated by dividing the number of spindles detected during N2 sleep by the total N2 sleep duration and averaged across both channels. If data from multiple nights were available, spindle density scores were then averaged across nights. For comparability with previous studies of spindles in BD [31], the current analyses will focus on slow and fast spindle density. A step-by-step visualisation of the sleep data processing can be found in supplement 1.

### Statistical analysis

Statistical analyses were performed in IBM SPSS Statistics (Version 31.0.0.0). Graphs were created in Rstudio (Version 4.2.2). The significance level was set at *α* = 0.05 for all analyses. We did not correct for multiple testing, as we only examined one primary outcome (correlation between fast spindle density and episodic memory composite score) and all other analyses were strictly exploratory and hypothesis-generating.

#### Cognitive performance

To calculate composite scores, individual test scores were transformed into z-scores using the mean and standard deviation from the current sample. The global cognition composite score was calculated as the sum of the z-scores for each individual test, divided by the number of tests. Similarly, the episodic memory composite score was calculated as the sum of the z-scores of the VPA1 and VPA2, divided by two. Scores from the TMT-A, TMT-B and Hotel Test were reversed (multiplied by −1) so that higher scores indicated better performance for all tests.

#### Correlation analyses

Partial Pearson correlation analysis with the covariate age was used to examine whether episodic memory performance assessed with the VPA1 and VPA2 was positively correlated with fast spindle density as our primary outcome. Exploratorily, partial correlations, controlling for age, between fast spindle density and all other cognitive tests (digit-symbol coding test, TMT-A, digit span test, FAS, hotel test, TMT-B, TOPF), the global cognition composite score and subjective sleep quality (PSQI), between slow spindle density and the global and episodic memory composite score and subjective sleep quality, as well as between subjective sleep quality and the global and episodic memory composite score, were investigated. Correlation analyses were repeated using non-parametric bootstrapping with 1000 resamples and percentile-based 95% confidence intervals.

#### Additional analyses

In a small sample, inclusion of multiple covariates may result in reduced statistical power and unstable estimates, which is why we only controlled for age in the main analyses. As gender and premorbid IQ may influence spindles and cognition, we additionally performed multiple linear regression models, adjusting for gender and premorbid IQ (TOPF score). Moreover, we conducted an unequal variance t-test and a chi-square test to examine differences in age, gender and premorbid IQ between those CRiB2 participants who participated in the EEG component (i.e., eligible and signed the EEG component informed consent form [ICF], but not necessarily analysed in the main EEG analyses) and those who did not (i.e., not eligible, not approached, not interested). Only participants who were ultimately included in the CRiB2 study (i.e., signed the CRiB2 ICF) could be included in this analysis, as we did not record any personal data of those who did not take part in the baseline assessment.

#### Sensitivity analyses

In sensitivity analyses, we repeated all analyses separately for participants who were medicated with lithium, and those who were not. To test for potential moderation effects, we then conducted moderation analyses using multiple linear regression models with fast spindle density as the predictor, and variables with significant correlations in the previous step as the outcome variable respectively, with lithium intake as the moderator, and age and gender as covariates. Fast spindle density was standardised (*M* = 0, *SD* = 1) and an interaction term between standardised fast spindle density and lithium intake computed. Additionally, an unequal variance t-test was performed to evaluate the differences in spindle density between both groups.

#### Post-hoc outlier sensitivity analysis

After visual inspection of the scatterplot of the primary outcome, we conducted a post-hoc sensitivity analysis excluding all visual outliers.

### Changes to the pre-registration

The pre-registered analysis plan stated that participants could only be included if they had not undergone trans-meridian travel within the past four weeks. This was changed to the past two weeks, in line with previous work [31]. In light of reduced sample size, fewer analyses were ultimately undertaken following protocol registration (see supplement 2 for changes in analyses).

## Results

### Participant characteristics

**Fig. 1:**
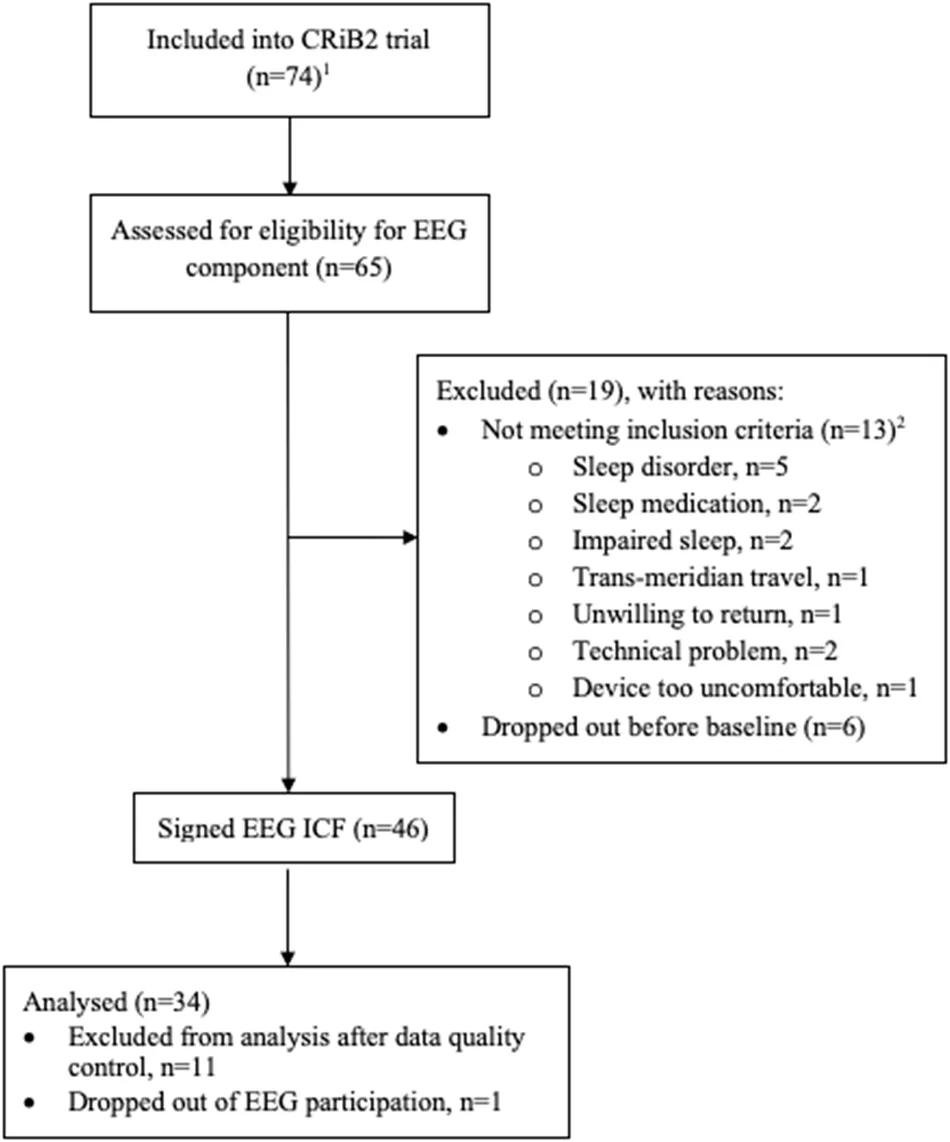
Participant in- and exclusion flowchart. *Note*. ^1^ Included into CRiB2 trial in London (from December 2023) and Oxford (from February 2024); ^2^ reasons do not add up to total amount of excluded participants, as multiple reasons could be applicable for exclusion; ICF: Informed consent form.

From the time of the EEG component commencement at the London and Oxford site, 74 participants were recruited for the CRiB2 trial. Sixty-five people were assessed for eligibility for the EEG component in the CRiB2 study (see Fig. 1). Nineteen people were excluded because they did not meet the inclusion criteria (see Fig. 1 for specific reasons) and seven people were ultimately not included in the CRiB2 study. Thus, 46 participants signed the ICF for the EEG component. A total of 34 participants were included into the final analysis; for 11 participants, none of the nights passed the quality control check (see EEG data preprocessing and quality control) and one participant dropped out of the EEG data collection after signing the ICF. Participants who participated in the EEG component (i.e., eligible and signed the EEG component ICF, not necessarily analysed in the main EEG analyses) were significantly younger (*M* = 42.3, *SD* = 10.89) than those who did not (i.e., not eligible, not approached, not interested) (*M* = 49.14, *SD* = 10.61; *p* = 0.010). Gender and premorbid IQ did not differ between both groups (see supplement 3).

A summary of the characteristics of the analysed participants can be found in Table 1. The majority of participants were white (76.5%), female (64.7%), had a BD-I diagnosis (58.8%) and have been diagnosed for *M* = 8.5 years. Participants were between 19 and 64 years old (*M* = 40.85, *SD* = 11.31), currently euthymic (HAMD and YMRS range: 0–6), had poor subjective sleep quality according to the PSQI [52], and an average premorbid IQ. All participants were medicated.

**Table 1:** Participant characteristics.

Demographic characteristics	Participants (*n* = 34)
Gender (*n, %*)	
Male	12 (35.3)
Female	22 (64.7)
Ethnicity (*n, %*)	
White British	21 (61.8)
Black British	1 (2.9)
Asian British	1 (2.9)
White Other	5 (14.7)
Black Other	1 (2.9)
Mixed	3 (8.8)
Other	2 (5.9)
Age (*M, SD)*	40.85 (11.31)
Type of BD (*n, %*)	
BD-I	20 (58.8)
BD-II	14 (41.2)
Years since BD diagnosis (*M, SD)*	8.5 (7.43)
Mood (*M, SD)*	
HAMD	2.6 (1.8)
YMRS	1.1 (1.7)
Number of medication (*M, SD)*	3.5 (3)
Currently under medication (*n, %*)^a^	34 (100)
Antidepressant	10 (29.4)
Mood stabiliser (excl. Lithium)	19 (55.9)
Lithium	12 (35.3)
Antipsychotic	20 (58.8)
Anxiolytic^b^	4 (11.8)
Hypnotic	0
Other – mental health related	2 (5.9)
General health	14 (41.2)
Subjective sleep quality PSQI (*M, SD)*	6.09 (3.43)
TOPF premorbid IQ (*M, SD)*	109.7 (8.37)

*HAMD*, Hamilton Rating Scale for Depression; *PSQI*, Pittsburgh Sleep Quality Index; *TOPF*, Test of Premorbid Functioning; *YMRS*, Young Mania Rating Scale.

^a^as per exclusion criterion, excluding melatonin, benzodiazepines, zopiclone, or zolpidem on a regular basis.

^b^anxiolytics included diazepam, promethazine, and lorazepam. All participants reported to take them on an irregular basis.

### Recordings and sleep characteristics

In total, 72 usable nights were collected and a mean of 2.12 (*SD* = 0.88) usable nights were collected per analysed participant. On average, 91.35% (*SD* = 8.71%) of epochs were usable per recording across participants and 92.3% (*SD* = 9.22%) across all recordings. Participants spent on average 192.82 min in N2 sleep when first averaged across recordings within participants and then across participants, and 199.63 min when averaged across all recordings irrespective of participant. Fast and slow spindle density across both channels for all nights correlated strongly and significantly with each other and fast and slow spindle density, averaged across channels, also correlated strongly and significantly across nights (see supplement 4 and 5 for correlation matrices).

### Sleep spindles

Table 2 displays descriptives for sleep spindle density, frequency, amplitude, and duration averaged across channels and nights. These were largely comparable with previous sleep spindle parameters obtained with standard polysomnography (PSG) [57] and Zmax devices [43].

**Table 2:** Spindle characteristics.

	Minimum	Maximum	Mean	Std. Deviation
**Fast spindle density** (spindles/min)	0.01	2.42	0.44	0.48
**Fast spindle frequency** (Hz)	13.46	13.84	13.67	0.1
**Fast spindle amplitude** (μV)	24.24	62.51	37.65	10.48
**Fast spindle duration** (seconds)	0.66	1.17	0.87	0.1
**Slow spindle density** (spindles/min)	0.12	5.4	2.32	1.53
**Slow spindle frequency** (Hz)	11.13	12.5	11.84	0.35
**Slow spindle amplitude** (μV)	26.02	65.93	40.07	10.78
**Slow spindle duration** (seconds)	0.76	1.3	0.96	0.134

### Sleep spindle-cognition correlations

All conditions for a partial Pearson correlation were met, except for the condition of normal distribution. However, the Pearson correlation is quite robust against this violation, and this condition can usually be disregarded if N > 30 [58]. Nonetheless, we repeated all main analyses with Spearman’s Rho, which resulted in largely the same results (see supplement 6). A full table with all Pearson correlations between all cognitive and sleep spindle parameters and zero-order correlations can be found in supplement 7, and scatterplots for all correlations in supplement 8.

#### Primary outcome: Fast spindle density and composite memory score

**Fig. 2:**
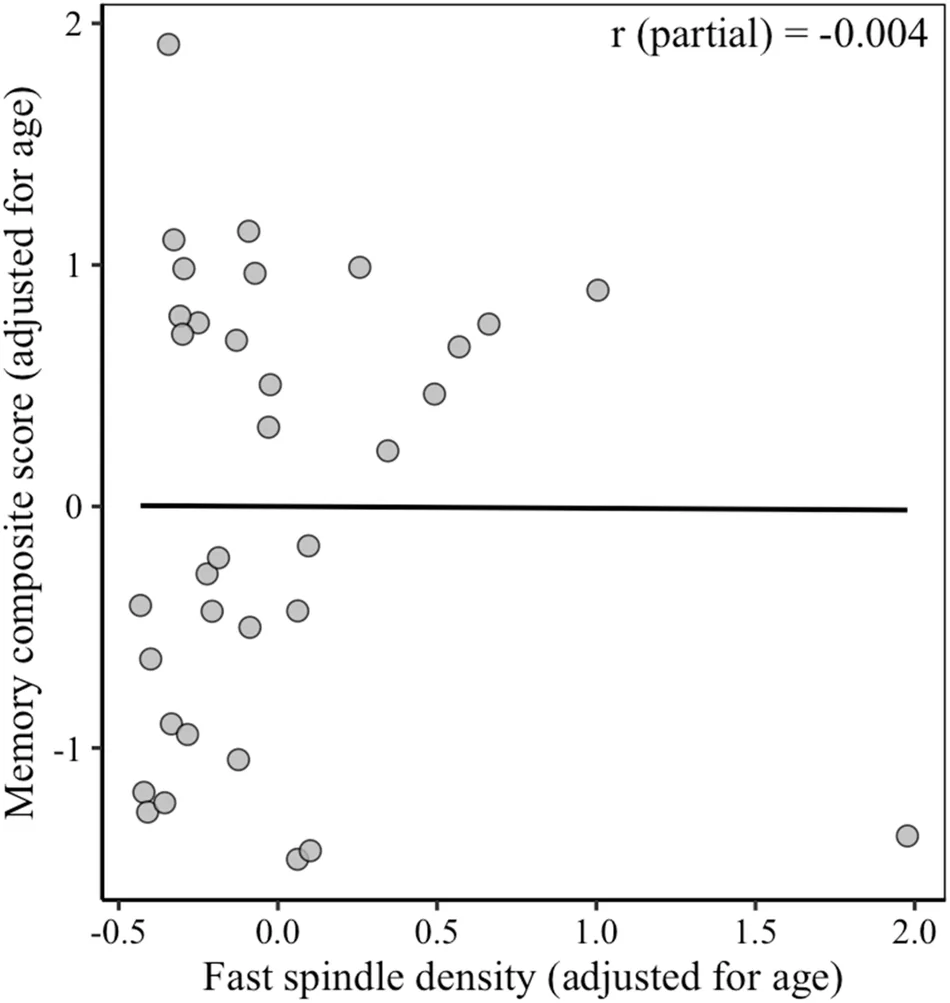
Scatterplot for fast spindle density and memory composite score adjusted for age. Note. Scatterplot for the primary outcome. The *x*-axis depicts the fast spindle density; the *y*-axis depicts the memory composite score, both controlled for age.

Table 3 reports on sleep spindle-cognition correlations and supplement 9 on bootstrapped 95% confidence intervals. Fast spindle density and episodic memory performance assessed with the VPA1 and VPA2 were not significantly associated with each other when controlling for age (*r* = −0.004, *p* = 0.491, 95% CI [−0.353, 0.499]; Fig. 2).

**Table 3:** Pearson correlation coefficients controlled for age.

	Fast spindle density	Slow spindle density	PSQI
**Memory composite score**	−0.004	0.173	−0.311
**Global cognition composite score**	0.300	0.191	−0.097
**PSQI**	0.009	−0.286	
**TOPF IQ**	0.085		
**Digit-symbol coding test**	0.057		
**Trail Making test A**	0.214		
**Digit span**	0.423*		
**FAS**	0.300		
**Hotel test**	0.232		
**Trail making test B**	0.126		

Only correlations specified in the aims are reported here, see supplement 7 for full correlation table; all correlations were controlled for age; all cognitive task scores are z-scores with higher values corresponding to better performance; spindle density was measured as spindles per minute; * indicates *p* < 0.05; 2-tailed correlation was calculated except for primary outcome (1-tailed, memory composite score and fast spindle density).

*FAS*, FAS letter fluency test; *PSQI*, Pittsburgh Sleep Quality Index; *TOPF*, Test of Premorbid Functioning.

#### Other exploratory spindle-cognition correlations

**Fig. 3:**
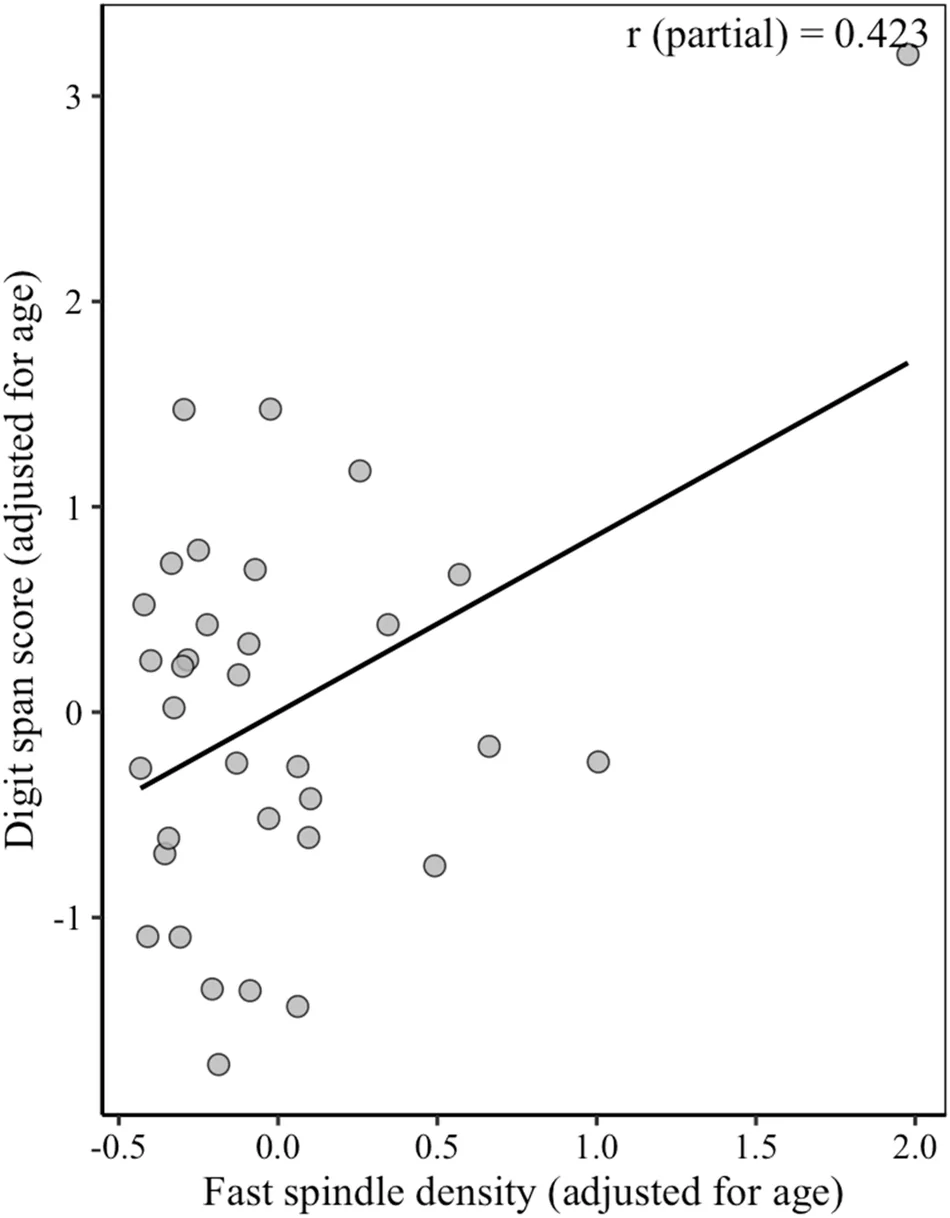
Scatterplot for fast spindle density and digit span score adjusted for age. Note. Scatterplot for the significant exploratory outcome. The *x*-axis depicts the fast spindle density; the *y*-axis depicts the working memory score, both controlled for age.

Fast spindle density correlated significantly positively with the digit span test (*r* = 0.423, *p* = 0.014, 95% CI [−0.167, 0.722]), indicating that more fast spindles per minute were associated with better performance (Fig. 3). However, it has to be noted that while the fast spindle-digit span correlation reached significance using standard *p*-values, its bootstrapped confidence interval crossed zero, suggesting that this effect may be less robust and should be interpreted with caution. Most exploratory correlations were non-significant (Table 3) (i.e., fast spindle density with all other cognitive tests [TOPF, digit-symbol coding test, TMT-A, FAS, hotel test, TMT-B], the global cognition composite score and subjective sleep quality [PSQI]; slow spindle density with the global and episodic memory composite score and subjective sleep quality; subjective sleep quality with the global and episodic memory composite score, all controlling for age).

#### Multiple linear regressions

The multiple linear regression models, adjusting for age, gender, and premorbid IQ (TOPF score) revealed the same results as the partial Pearson correlations (see supplement 10). Fast spindle density significantly predicted digit span performance (*b* = 0.857, 95% CI [0.184, 1.531], *β* = 0.419, *p* = 0.014), but findings for all other outcome variables were non-significant (i.e. memory composite score, global cognition composite score, PSQI, digit-symbol coding test, TMT-A, FAS, Hotel test, TMT-B), as for slow spindle density and subjective sleep quality (PSQI score).

#### Lithium sensitivity analysis

Twelve participants were medicated with lithium, and 22 were not. While all partial correlations for lithium-treated participants were non-significant, for those participants who were not treated with lithium, fast sleep spindle density was significantly positively associated with global cognition (*r* = 0.441, *p* = 0.045, 95% CI [−0.016, 0.672]), the digit span test (*r* = 0.671, *p* < 0.001, 95% CI [−0.118, 0.897]), and the FAS (*r* = 0.548, *p* = 0.010, 95% CI [−0.198, 0.836]), with a higher fast spindles count per minute indicating better performance. Similarly to the main analyses, however, the bootstrapped confidence intervals crossed zero for all associations, indicating that these results must be interpreted with caution. Subjective sleep quality was negatively associated with the composite memory score (*r* = −0.529, *p* = 0.014, 95% CI [−0.774, −0.154]), meaning worse subjective sleep quality was associated with worse memory performance (see Table 4 and supplement 11 for bootstrapped confidence intervals). The moderation analyses revealed no moderation effect of lithium on the associations between fast spindle density and global cognition, the digit span test or the FAS, adjusting for age and gender (see supplement 12). However, fast spindle density significantly predicted all three outcome variables in these moderation models (global cognition: *b* = 0.266, 95% CI [0.033, 0.498], *β* = 0.481, *p* = 0.026; digit span: *b* = 0.694, 95% CI [0.326, 1.063], *β* = 0.694, *p* < 0.001; FAS: *b* = 0.555, 95% CI [0.163, 0.946], *β* = 0.555, *p* = 0.007), suggesting that higher fast spindle density is associated with better global cognition, as well as digit span and FAS performance, when adjusting for lithium intake, age, gender and the fast spindle density x lithium interaction. Participants treated with lithium had higher fast and slow spindle density (lithium treated: fast: *M* = 0.52, *SD* = 0.45; slow: *M* = 2.83, *SD* = 1.58; not lithium treated: fast: *M* = 0.4, *SD* = 0.51; slow: *M* = 2.04, *SD* = 1.47), although the differences were not statistically significant (fast: *t*(25.32) = 0.7, *p* = 0.492; slow: *t*(21.44) = 1.43, *p* = 0.167).

**Table 4:** Pearson correlation coefficients controlled for age by lithium intake.

	Fast spindle density		Slow spindle density		PSQI	
*Lithium*	*no*	*yes*	*no*	*yes*	*no*	*yes*
**Memory composite score**	−0.223	0.509	0.056	0.409	−0.529*	0.172
**Global cognition composite score**	0.441*	−0.056	0.429	−0.250	−0.263	0.203
**PSQI**	−0.041	0.263	−0.352	−0.030		
**TOPF IQ**	0.242	−0.256				
**Digit-symbol coding test**	0.378	−0.584				
**Trail Making test A**	0.310	−0.062				
**Digit span**	0.671**	−0.075				
**FAS**	0.548*	−0.370				
**Hotel test**	0.314	0.090				
**Trail making test B**	0.353	−0.210				

Only correlations specified in the aims are reported here; all correlations were controlled for age; all cognitive task scores are z-scores with higher values corresponding to better performance; spindle density was measured as spindles per minute; * indicates *p* < 0.05, ** indicates *p* < 0.01.

*FAS*, FAS letter fluency test; *PSQI*, Pittsburgh Sleep Quality Index; *TOPF*, Test of Premorbid Functioning.

#### Post-hoc outlier sensitivity analysis

When excluding the two visual outliers evident in Fig. 2 from the primary analysis, the correlation was positive and significant (*r* = 0.368, *p* = 0.021, 95% CI [0.026, 0.611]).

### Deviation from the pre-registration and adverse events

The pre-registration stated that participants were to record their sleep within the first week after the baseline assessment. However, six of the analysed participants provided data within two to three weeks after the baseline assessment, whom were retained to maximise power. Adverse events were recorded systematically at the end of every CRiB2 assessment (baseline, post, and follow-up). Please note that only the adverse events reported at the baseline assessment are reported here, as data recorded at post and follow-up was not used for the present analysis. One participant reported panic-like symptoms caused by the timer going off during the hotel test. Four participants reported emotional stress caused by some of the questionnaires, particularly clinical screeners, employed during the assessment. Lastly, one participant reported transient stress during the cognitive tasks.

Regarding the EEG component, adverse events specifically for the EEG component were *not* systematically recorded immediately after EEG data collection, so we only report adverse events that participants reported without being prompted to. However, participants could have reported any adverse events that happened during EEG data collection at the post- or follow-up assessment, which did not occur. Immediately after the EEG return, three participants reported impaired sleep due to the EEG headband being uncomfortable and three participants reported a temporary mark on their forehead caused by the EEG headband. All participants agreed to continue the study despite the adverse events reported and had recovered at study end.

## Discussion

To the best of our knowledge, this is the first study to investigate the relationship between fast spindle density and episodic memory in BD, as well as relationships between fast and slow spindle density, several cognitive performance scores, and subjective sleep quality. Contrary to our hypothesis, in a sample of 34 euthymic participants with BD, our primary outcome was not significant, with no significant relationship between fast spindle density and episodic memory (composite memory score comprised of the VPA1 and VPA2). However, several exploratory outcomes were significant; more fast spindles per minute were associated with a better working memory performance and, when splitting the sample into participants with and without lithium intake, there were several additional significant associations for those not taking lithium, and fast and slow spindle densities were non-significantly higher for those taking lithium.

The finding that there was no significant correlation between fast spindle density and episodic memory was somewhat unexpected. There are multiple pathways for how fast spindles may facilitate memory consolidation. Sleep spindles are generated in the thalamic reticular nucleus (TRN) and transmitted to the cortex through the thalamocortical circuit, where they increase glutamate receptor expression and create an environment suitable for long-term potentiation. Synchronisation with hippocampal sharp-wave ripples by slow oscillations facilitates hippocampal-neocortex transfer of information [59]. Moreover, sleep spindles promote synaptic plasticity and contribute to sensory gating during sleep, shielding NREM sleep from sensory or spontaneous disruption [17]. Further, there is robust evidence for spindle deficits in schizophrenia with fairly consistent links to memory performance. In healthy first-degree relatives of patients with schizophrenia, Schilling et al. [19] identified a significant association between verbal learning and memory and fast spindle density and Göder et al. [25] found that sleep spindle density was positively associated with better visual memory in schizophrenia. However, other studies did not identify significant correlations between spindle density and specific memory tests [24, 26, 28]. We found no correlation for fast spindle density and episodic memory/verbal learning. However, in a post-hoc exploratory sensitivity analysis, excluding two visual outliers, we identified a positive, significant association between fast spindle density and episodic memory. In further exploratory analyses, we found a positive correlation between fast spindle density and working memory and a possible influence of lithium on these effects.

One explanation for the heterogeneous results could be the different tests employed. It is possible that only specific tests tap into cognitive domains associated with spindle processes, although some of the tests with contrasting results measure virtually the same domain. Alternatively, Au and Harvey’s [18] meta-analysis of sleep spindle activity and cognitive function in psychosis pointed to the methodological variability in sleep-EEG acquisition, sleep-stage scoring, and spindle-detection techniques and underpowered analyses as a likely source of the inconsistent results reported across studies. Similarly, although our sample size was larger than in most previous studies, we did not achieve our planned sample size of 36 and our analysis might have been underpowered. Our post-hoc outlier sensitivity analysis further highlights the impact of extreme values in smaller samples, as the primary outcome was significant when excluding two outliers. Further, it is possible that correlations significant in samples with schizophrenia do not uphold in BD samples. Sleep spindle deficits in schizophrenia have been linked to a dysfunctional thalamocortical circuit, with reduced connectivity between thalamus and prefrontal cortex [60] and reduced thalamic volumes [61] and increased connectivity between the thalamus and sensorimotor cortex [62] being associated with decreased sleep spindle activity and cognitive performance. In BD, findings are more heterogeneous and linkages have only been partially documented so far; there are documented effects of lithium [63] and mood states [64] on thalamus volumes and dysconnectivity patterns within thalamocortical systems in BD that are qualitatively different to schizophrenia, while BD patients with psychosis history exhibit patterns more consistent with schizophrenia [65]. Further, there is shared neural evidence for TRN abnormalities in both schizophrenia and BD [66] and a genetic link between fast spindle density and the polygenic score for schizophrenia, which is, however, less pronounced for BD [67]. Ritter et al. [31] examined sleep spindle parameters in 23 euthymic BD patients compared to 25 healthy controls and found a significantly lower fast spindle (>13 Hz) density in BD compared to healthy controls. Thus, it seems while mechanisms could largely overlap resulting in spindle abnormalities across disorders, pathways could be less pronounced or more heterogeneous in BD and specific medications, like lithium, could have attenuating effects.

Notably, in our exploratory analyses limited to participants not taking lithium, higher fast spindle density showed significant associations with global cognition, digit span, and the FAS. In addition, poorer subjective sleep quality was associated with a poorer composite memory score. Further, fast spindle density was significantly associated with global cognition, digit span, and the FAS when controlling for age, gender, and lithium intake, although without a moderation effect of lithium status. These patterns raise the possibility that lithium or factors associated with lithium use may influence spindle-cognition relationships. While evidence is inconsistent, some data suggest pro-cognitive effects of lithium across a wide range of domains in BD [33]. Additionally, lithium seems to modulate the circadian clock [34], sleep architecture [68] and thalamus volumes (see above; [63]), and contributes to better sleep efficiency and fewer sleep disturbances compared to valproic acid in BD [69]. Both the present investigation, and previously Ritter et al. [31] have found non-significantly higher spindle densities in participants taking lithium. Although we can only speculate about the specific pathways through which lithium may dampen the relationship between fast spindle density and cognitive performance, it possibly nonlinearly enhances cognitive performance and spindle density.

### Limitations

There are several important limitations to consider. Firstly, our findings are correlative, so it is not possible to draw strong conclusions about causal relationships between spindles and cognitive performance, and most analyses were exploratory in nature, so results should be considered hypothesis-generating. Additionally, while the standard *p*-values for the fast spindle density–working memory correlation, as well as several other correlations in the lithium sensitivity analysis, reached significance and the multiple linear regression analyses, additionally adjusting for gender and premorbid IQ (and lithium in the sensitivity analyses), also supported these associations, the bootstrapped confidence intervals crossed zero. This suggests that these findings may be sensitive to sampling variability and should be interpreted with caution. Secondly, we did not reach our intended sample size of 36, which was calculated to detect a correlation of *r* = 0.45. With a final sample size of 34, our study may therefore have been underpowered to detect small-to-moderate associations, and all observed correlations were indeed smaller than *r* = 0.45 in the main analyses. In addition, most correlations were in the expected direction, which may suggest that true, albeit small, associations were not detected due to limited statistical power. Thirdly, in contrast to PSG setups, our device only comprised two frontal electrodes. Considering that sleep spindles can be found across the majority of the cortical surface, with fast spindles mainly presenting in centroparietal and slow spindles more globally and in more anterior regions [17], it is likely that we have missed spindle events in some areas. This might also explain why we identified higher slow (*M* = 2.32) than fast spindle densities (*M* = 0.44); although our overall spindle density (*M* = 2.76) was higher than in a major sleep spindle analysis of 11630 individuals with standard PSG (*M* = 1.88) [57]. Fourthly, there are additional concerns about the validity and reliability of mobile sleep-EEG devices, e.g., the lack of electrooculography (EOG) and electromyography (EMG) or the potentially incorrect positioning of the headband. Yet, a validation study by Esfahani et al. [43] concluded that Zmax can produce reliable sleep metrics often comparable to standard PSG. Additionally, while a distinction between fast and slow spindles at 13 Hz is common, other fixed cut-offs or spindle detection methods adjusting for individual spindle frequency differences would have been possible and might have resulted in different results. Moreover, everyone in our sample was taking psychotropic medication. As medication effects and doses interact in a complex manner, we were unable to control for their effects on sleep and cognition, apart from the lithium sensitivity analyses. However, participants taking medications known to have a direct effect on sleep spindles were excluded when taken on a regular basis (zopiclone or zolpidem; “z-drugs”) [70] and excluding further medication might have resulted in a non-representative sample. Furthermore, we did not exclude the first night from our analyses, which might have induced a first-night effect. However, as the mobile EEG-device is less invasive than standard PSG set-ups and participants were sleeping in their own homes, this effect was likely attenuated. Participants were also not instructed to modify their behaviour with regard to adherence to sleep hygiene guidelines, caffeine, recreational substance (e.g., cannabis or nicotine) or alcohol intake, or exercise. Although this may have increased variability, it also likely improved ecological validity, especially as participants were free to maintain their usual sleep-wake schedules. Additionally, our results may not be generalisable to the wider BD population, as participants with specific comorbidities (e.g., sleep disorders, neurological or inflammatory conditions) were excluded and our sample was mainly white and female. Moreover, participants in the EEG component were significantly younger than those who did not participate, though we controlled for age in all our analyses. Further, the time of day for cognitive assessments was not standardised, but as participants were free to choose a time convenient for them, they may have selected a time when they felt more cognitively able (although free-time from professional and personal commitments most likely also played a role). Moreover, we did not systematically record adverse events of the EEG component immediately after EEG return, so some adverse events might have been missed; however, as they were systematically recorded at post- and follow-up assessments, participants could have reported them then, which was not the case. Lastly, we did not have a control group, preventing spindle characteristic or correlation comparisons with healthy controls or other populations, e.g., first-degree relatives or participants with psychosis.

### Implications and future directions

Future studies with larger sample sizes, longitudinal designs and control groups could illuminate more specific interactions between sleep spindles and cognitive performance, e.g., the influence of mood, medication (e.g., lithium) or illness progression, different spindle parameters (e.g., amplitude, frequency, duration), or differences between subsamples, also in combination with therapy, like psychotherapy (e.g., CRT) or pharmacotherapy. With more comprehensive evidence, sleep spindles could serve as a candidate endophenotype, and, for example, be used as a tool for early recognition of BD or psychosis. Sleep spindles have even been suggested as a potential target for cognitive enhancement: sleep spindles can be detected in real time with closed-loop feedback strategies and suppressed or boosted according to the hypothesized function [17]. Moreover, meta-analytic evidence suggests that z-drugs show therapeutic potential to enhance sleep-dependent memory consolidation by increasing sleep spindle activity [70]. Overall, sleep spindles bear the potential for early disorder recognition, as well as better understanding and treatment of cognitive difficulties. Using new methods of sleep recording and analysis, like mobile EEG devices and fully automated sleep staging and spindle detection like in the present study will likely be on the forefront of this endeavour, as it enables sleep research on a larger scale.

## Conclusion

The current study was unable to identify an association between fast spindle density and episodic memory in euthymic bipolar patients. In uncorrected, exploratory analyses, preliminary evidence for an association between fast spindle density and working memory, but not for slow spindles and other cognitive domains, was found. Sensitivity analyses suggested that lithium may influence the relationship between sleep spindles and cognitive performance. Future large-scale studies are needed to deepen our understanding of these associations, and mobile sleep devices with automatic sleep-stage and spindle detection will likely serve as valuable tools in this effort.

## Supplementary information


Supplementary Material


## Data Availability

The data that support the findings of this study are available from the corresponding author upon reasonable request.
