# Supplementary material for: The association between sleep spindles and cognitive performance in euthymic bipolar disorder
Source: Transl Psychiatry. 2026 Jul 14;16:434. doi: 10.1038/s41398-026-04273-2 (PMC13524930; doi:10.1038/s41398-026-04273-2)
Supplement: Supplementary file 1 [file 41398_2026_4273_MOESM1_ESM.docx]

**Supplementary Material**

**Supplement 1: Visualisation of the EEG data processing procedure**

*Note.* Created in BioRender. Maicher, B. (2026) https:// BioRender.com/p5bv0lj”; IQR – interquartile range.

**Supplement 2: Changes to the analyses following pre-registration**

The pre-registration stated the correlations between fast spindle density and cognition (global cognition composite score and individual cognitive domain scores (processing speed and attention, working memory, verbal learning, phonetic and verbal fluency, memory, and executive function)) as the primary outcomes. Associations between other fast spindle characteristics (frequency, amplitude, duration, and coherence) as well as subjective sleep quality and cognition were registered as secondary outcomes. Associations with other sleep parameters (e.g., N3 sleep percentage) with cognition were registered as exploratory analyses. Instead, we only focused on the correlation between a memory composite score with fast spindle density as a primary outcome, and correlations between fast spindle density with the global cognition score and other individual test scores, between slow spindle density with the memory and global composite score, and with subjective sleep quality, as exploratory outcomes. This decision was made before any data analyses had started and to provide a more comprehensive and hypothesis-driven report. Because only one primary outcome was chosen, no false discovery rates (FDR) correction for multiple testing was performed like stated in the pre-registration.

**Supplement 3: Sociodemographic differences between EEG participants and non-participants**

|  | **EEG participants** (n=46) | **EEG non-participants**  (n=28) | **Test statistics** |
| --- | --- | --- | --- |
| **Age** (*M, SD*) | 42.3 (10.89) | 49.14 (10.61) | *t*(58)=2.66, ***p*=.010*** |
| **Gender** (n, %) |  |  | χ²(1) = 0.002, p = .967 |
| Male | 15 (32.6) | 9 (32.1) |  |
| Female | 31 (67.4) | 19 (67.9) |  |
| **TOPF**(*M, SD*) | 109.37 (8.67) | 111.64 (8.12) | *t*(60)=1.14, *p*=.259 |

*Note.* EEG participants refer to people who were eligible and signed the EEG component informed consent form, but not necessarily analysed in the main EEG analyses; EEG non-participants refer to people who were not eligible, not interested, or not approached. People who dropped out of the CRiB2 study before the baseline assessment could not be included into this analysis; TOPF – Test of Premorbid Functioning.

**Supplement 4: Correlations of fast and slow spindle density across channels**

|  | | FSD R night 1 | SSD R night 1 | FSD R night 2 | SSD R night 2 | FSD R night 3 | SSD R night 3 | FSD R night 4 | SSD R night 4 |
| --- | --- | --- | --- | --- | --- | --- | --- | --- | --- |
| FSD L night 1 | Pearson Correlation | .972^**^ | .563^**^ | .923^**^ | .659^**^ | .736^**^ | .730^**^ | .^c^ | .^c^ |
|  | Sig. (2-tailed) | <.001 | .002 | <.001 | .004 | .001 | .001 | . | . |
|  | N | 28 | 28 | 17 | 17 | 16 | 16 | 0 | 0 |
| SSD L night 1 | Pearson Correlation | .580^**^ | .956^**^ | .509^*^ | .861^**^ | .256 | .799^**^ | .^c^ | .^c^ |
|  | Sig. (2-tailed) | .001 | <.001 | .037 | <.001 | .338 | <.001 | . | . |
|  | N | 28 | 28 | 17 | 17 | 16 | 16 | 0 | 0 |
| FSD L night 2 | Pearson Correlation | .971^**^ | .533^*^ | .876^**^ | .595^**^ | .893^**^ | .794^**^ | .^c^ | .^c^ |
|  | Sig. (2-tailed) | <.001 | .028 | <.001 | .006 | <.001 | <.001 | . | . |
|  | N | 17 | 17 | 20 | 20 | 16 | 16 | 1 | 1 |
| SSD L night 2 | Pearson Correlation | .590^*^ | .906^**^ | .519^*^ | .860^**^ | .385 | .869^**^ | .^c^ | .^c^ |
|  | Sig. (2-tailed) | .013 | <.001 | .019 | <.001 | .141 | <.001 | . | . |
|  | N | 17 | 17 | 20 | 20 | 16 | 16 | 1 | 1 |
| FSD L night 3 | Pearson Correlation | .622^*^ | .351 | .543^*^ | .405 | .915^**^ | .742^**^ | .984 | .963 |
|  | Sig. (2-tailed) | .010 | .182 | .030 | .119 | <.001 | <.001 | .116 | .174 |
|  | N | 16 | 16 | 16 | 16 | 21 | 21 | 3 | 3 |
| SSD L night 3 | Pearson Correlation | .518^*^ | .560^*^ | .374 | .653^**^ | .616^**^ | .903^**^ | .998^*^ | .987 |
|  | Sig. (2-tailed) | .040 | .024 | .154 | .006 | .003 | <.001 | .044 | .103 |
|  | N | 16 | 16 | 16 | 16 | 21 | 21 | 3 | 3 |
| FSD L night 4 | Pearson Correlation | .^c^ | .^c^ | .^c^ | .^c^ | .994 | .963 | .962 | .932 |
|  | Sig. (2-tailed) | . | . | . | . | .069 | .175 | .177 | .235 |
|  | N | 0 | 0 | 1 | 1 | 3 | 3 | 3 | 3 |
| SSD L night 4 | Pearson Correlation | .^c^ | .^c^ | .^c^ | .^c^ | .987 | 1.000^**^ | 1.000^**^ | .995 |
|  | Sig. (2-tailed) | . | . | . | . | .101 | .004 | .006 | .065 |
|  | N | 0 | 0 | 1 | 1 | 3 | 3 | 3 | 3 |

*Note.* **Correlation is significant at the 0.01 level (2-tailed); *Correlation is significant at the 0.05 level (2-tailed); ^c^ cannot be computed because at least one of the variables is constant; FSD – fast spindle density; SSD – slow spindle density; L – left channel (F7); R- right channel (F8).

**Supplement 5: Correlations of fast and slow spindle density, averaged across channels, across nights**

|  | | FSD night 1 | SSD night 1 | FSD night 2 | SSD night 2 | FSD night 3 | SSD night 3 | FSD night 4 | SSD night 4 |
| --- | --- | --- | --- | --- | --- | --- | --- | --- | --- |
| FSD night 1 | Pearson Correlation | 1 | .588^**^ | .977^**^ | .652^**^ | .755^**^ | .696^**^ | .^b^ | .^b^ |
|  | Sig. (2-tailed) |  | <.001 | <.001 | .005 | <.001 | .003 | . | . |
|  | N | 28 | 28 | 17 | 17 | 16 | 16 | 0 | 0 |
| SSD night 1 | Pearson Correlation | .588^**^ | 1 | .537^*^ | .923^**^ | .330 | .721^**^ | .^b^ | .^b^ |
|  | Sig. (2-tailed) | <.001 |  | .026 | <.001 | .212 | .002 | . | . |
|  | N | 28 | 28 | 17 | 17 | 16 | 16 | 0 | 0 |
| FSD night 2 | Pearson Correlation | .977^**^ | .537^*^ | 1 | .653^**^ | .815^**^ | .653^**^ | .^b^ | .^b^ |
|  | Sig. (2-tailed) | <.001 | .026 |  | .002 | <.001 | .006 | . | . |
|  | N | 17 | 17 | 20 | 20 | 16 | 16 | 1 | 1 |
| SSD night 2 | Pearson Correlation | .652^**^ | .923^**^ | .653^**^ | 1 | .459 | .851^**^ | .^b^ | .^b^ |
|  | Sig. (2-tailed) | .005 | <.001 | .002 |  | .073 | <.001 | . | . |
|  | N | 17 | 17 | 20 | 20 | 16 | 16 | 1 | 1 |
| FSD night 3 | Pearson Correlation | .755^**^ | .330 | .815^**^ | .459 | 1 | .739^**^ | .999^*^ | .977 |
|  | Sig. (2-tailed) | <.001 | .212 | <.001 | .073 |  | <.001 | .025 | .138 |
|  | N | 16 | 16 | 16 | 16 | 21 | 21 | 3 | 3 |
| SSD night 3 | Pearson Correlation | .696^**^ | .721^**^ | .653^**^ | .851^**^ | .739^**^ | 1 | .995 | .997^*^ |
|  | Sig. (2-tailed) | .003 | .002 | .006 | <.001 | <.001 |  | .066 | .047 |
|  | N | 16 | 16 | 16 | 16 | 21 | 21 | 3 | 3 |
| FSD night 4 | Pearson Correlation | .^b^ | .^b^ | .^b^ | .^b^ | .999^*^ | .995 | 1 | .984 |
|  | Sig. (2-tailed) | . | . | . | . | .025 | .066 |  | .113 |
|  | N | 0 | 0 | 1 | 1 | 3 | 3 | 3 | 3 |
| SSD night 4 | Pearson Correlation | .^b^ | .^b^ | .^b^ | .^b^ | .977 | .997^*^ | .984 | 1 |
|  | Sig. (2-tailed) | . | . | . | . | .138 | .047 | .113 |  |
|  | N | 0 | 0 | 1 | 1 | 3 | 3 | 3 | 3 |
| *Note.* **. Correlation is significant at the 0.01 level (2-tailed); *Correlation is significant at the 0.05 level (2-tailed); b. Cannot be computed because at least one of the variables is constant; FSD – fast spindle density; SSD – slow spindle density. | | | | | | | | | |
|  | | | | | | | | | |
|  | | | | | | | | | |

**Supplement 6: Spearman’s Rho coefficients controlled for age**

| Control Variables | | | Memory composite score | Global cognition composite score |
| --- | --- | --- | --- | --- |
| age | Fast spindle density | Correlation | .097 | .174 |
|  |  | Significance (2-tailed) | .591 | .332 |
|  | Slow spindle density | Correlation | .171 | .186 |
|  |  | Significance (2-tailed) | .341 | .299 |

**Supplement 7: Pearson correlation coefficients with and without controlling for age**

| Control |  |  | Global cognition composite score | Memory composite score | TOPF IQ | Digit-symbol coding test | Trail Making test A (reversed) | Digit span | Verbal Pair associates test I | FAS | Verbal Pair associates II | Hotel test (reversed) | Trail making test B (reversed) | PSQI |
| --- | --- | --- | --- | --- | --- | --- | --- | --- | --- | --- | --- | --- | --- | --- |
| / | PSQI | Correlation | -.100 | -.374 | .060 | -.079 | .176 | .183 | -.396 | .066 | -.333 | -.224 | .164 | 1.000 |
|  |  | Significance (2-tailed) | .574 | .029 | .737 | .658 | .319 | .300 | .021 | .712 | .055 | .203 | .354 | . |
|  |  | df | 32 | 32 | 32 | 32 | 32 | 32 | 32 | 32 | 32 | 32 | 32 | 0 |
|  | All spindle density | Correlation | .236 | .164 | -.008 | -.116 | .191 | .267 | .089 | .123 | .230 | .216 | .043 | -.250 |
|  |  | Significance (2-tailed) | .179 | .354 | .965 | .513 | .280 | .126 | .617 | .487 | .190 | .219 | .808 | .154 |
|  |  | df | 32 | 32 | 32 | 32 | 32 | 32 | 32 | 32 | 32 | 32 | 32 | 32 |
|  | All spindle frequency | Correlation | .081 | .134 | -.127 | -.184 | -.079 | .111 | .164 | .142 | .096 | .193 | -.086 | -.002 |
|  |  | Significance (2-tailed) | .650 | .452 | .475 | .299 | .657 | .533 | .353 | .424 | .591 | .274 | .628 | .990 |
|  |  | df | 32 | 32 | 32 | 32 | 32 | 32 | 32 | 32 | 32 | 32 | 32 | 32 |
|  | All spindle amplitude | Correlation | .163 | .120 | -.118 | .123 | .302 | .111 | .097 | -.027 | .136 | -.234 | .213 | -.230 |
|  |  | Significance (2-tailed) | .356 | .500 | .507 | .489 | .082 | .532 | .587 | .880 | .442 | .182 | .225 | .190 |
|  |  | df | 32 | 32 | 32 | 32 | 32 | 32 | 32 | 32 | 32 | 32 | 32 | 32 |
|  | All spindle duration | Correlation | -.018 | .139 | -.152 | -.275 | -.085 | .019 | .068 | -.028 | .204 | .138 | -.120 | -.306 |
|  |  | Significance (2-tailed) | .921 | .431 | .391 | .116 | .634 | .914 | .704 | .874 | .247 | .435 | .499 | .078 |
|  |  | df | 32 | 32 | 32 | 32 | 32 | 32 | 32 | 32 | 32 | 32 | 32 | 32 |
|  | Fast spindle density | Correlation | .300 | -.004 | .085 | .057 | .204 | .421 | -.036 | .298 | .028 | .230 | .126 | .009 |
|  |  | Significance (2-tailed) | .085 | .981 | .633 | .751 | .248 | .013 | .840 | .087 | .876 | .190 | .477 | .961 |
|  |  | df | 32 | 32 | 32 | 32 | 32 | 32 | 32 | 32 | 32 | 32 | 32 | 32 |
|  | Fast spindle frequency | Correlation | -.116 | .039 | -.030 | .075 | -.167 | -.204 | .029 | -.305 | .047 | -.196 | .208 | -.002 |
|  |  | Significance (2-tailed) | .513 | .827 | .867 | .673 | .345 | .246 | .871 | .079 | .793 | .266 | .237 | .991 |
|  |  | df | 32 | 32 | 32 | 32 | 32 | 32 | 32 | 32 | 32 | 32 | 32 | 32 |
|  | Fast spindle amplitude | Correlation | .117 | .035 | -.118 | .163 | .290 | .070 | .017 | -.015 | .051 | -.256 | .197 | -.194 |
|  |  | Significance (2-tailed) | .511 | .845 | .506 | .356 | .097 | .695 | .924 | .934 | .774 | .144 | .265 | .271 |
|  |  | df | 32 | 32 | 32 | 32 | 32 | 32 | 32 | 32 | 32 | 32 | 32 | 32 |
|  | Fast spindle duration | Correlation | .169 | .088 | -.004 | -.078 | .078 | .184 | .038 | .112 | .134 | .252 | .028 | -.226 |
|  |  | Significance (2-tailed) | .339 | .620 | .982 | .660 | .663 | .297 | .833 | .527 | .449 | .150 | .875 | .199 |
|  |  | df | 32 | 32 | 32 | 32 | 32 | 32 | 32 | 32 | 32 | 32 | 32 | 32 |
|  | Slow spindle density | Correlation | .193 | .202 | -.037 | -.160 | .168 | .193 | .120 | .056 | .272 | .191 | .013 | -.308 |
|  |  | Significance (2-tailed) | .275 | .253 | .837 | .366 | .343 | .275 | .499 | .754 | .119 | .280 | .943 | .076 |
|  |  | df | 32 | 32 | 32 | 32 | 32 | 32 | 32 | 32 | 32 | 32 | 32 | 32 |
|  | Slow spindle frequency | Correlation | .053 | .152 | -.191 | -.273 | -.107 | .116 | .178 | .146 | .119 | .196 | -.140 | -.047 |
|  |  | Significance (2-tailed) | .765 | .390 | .279 | .119 | .548 | .515 | .315 | .411 | .502 | .267 | .431 | .794 |
|  |  | df | 32 | 32 | 32 | 32 | 32 | 32 | 32 | 32 | 32 | 32 | 32 | 32 |
|  | Slow spindle amplitude | Correlation | .178 | .123 | -.109 | .126 | .310 | .130 | .099 | -.016 | .140 | -.225 | .222 | -.224 |
|  |  | Significance (2-tailed) | .315 | .487 | .540 | .478 | .075 | .464 | .576 | .927 | .428 | .200 | .207 | .203 |
|  |  | df | 32 | 32 | 32 | 32 | 32 | 32 | 32 | 32 | 32 | 32 | 32 | 32 |
|  | Slow spindle duration | Correlation | -.016 | .132 | -.161 | -.265 | -.086 | .032 | .060 | -.023 | .196 | .143 | -.131 | -.303 |
|  |  | Significance (2-tailed) | .927 | .458 | .364 | .130 | .630 | .857 | .735 | .897 | .266 | .420 | .460 | .081 |
|  |  | df | 32 | 32 | 32 | 32 | 32 | 32 | 32 | 32 | 32 | 32 | 32 | 32 |
| age | PSQI | Correlation | -.097 | -.311 | .055 | -.092 | .093 | .158 | -.340 | .028 | -.265 | -.205 | .173 | 1.000 |
|  |  | Significance (2-tailed) | .592 | .078 | .760 | .611 | .607 | .379 | .053 | .878 | .137 | .252 | .337 | . |
|  |  | df | 31 | 31 | 31 | 31 | 31 | 31 | 31 | 31 | 31 | 31 | 31 | 0 |
|  | All spindle density | Correlation | .235 | .140 | -.005 | -.114 | .235 | .282 | .063 | .139 | .210 | .208 | .043 | -.231 |
|  |  | Significance (2-tailed) | .188 | .436 | .976 | .528 | .189 | .112 | .728 | .441 | .241 | .244 | .811 | .195 |
|  |  | df | 31 | 31 | 31 | 31 | 31 | 31 | 31 | 31 | 31 | 31 | 31 | 31 |
|  | All spindle frequency | Correlation | .079 | .115 | -.125 | -.182 | -.057 | .121 | .148 | .154 | .074 | .187 | -.087 | .023 |
|  |  | Significance (2-tailed) | .663 | .526 | .488 | .311 | .753 | .503 | .412 | .391 | .681 | .298 | .632 | .899 |
|  |  | df | 31 | 31 | 31 | 31 | 31 | 31 | 31 | 31 | 31 | 31 | 31 | 31 |
|  | All spindle amplitude | Correlation | .162 | .109 | -.117 | .125 | .336 | .118 | .085 | -.020 | .126 | -.241 | .214 | -.225 |
|  |  | Significance (2-tailed) | .367 | .547 | .517 | .489 | .056 | .513 | .638 | .913 | .485 | .177 | .233 | .209 |
|  |  | df | 31 | 31 | 31 | 31 | 31 | 31 | 31 | 31 | 31 | 31 | 31 | 31 |
|  | All spindle duration | Correlation | -.022 | .093 | -.150 | -.274 | -.034 | .039 | .020 | -.005 | .161 | .124 | -.122 | -.271 |
|  |  | Significance (2-tailed) | .901 | .608 | .404 | .123 | .851 | .829 | .913 | .976 | .371 | .491 | .498 | .127 |
|  |  | df | 31 | 31 | 31 | 31 | 31 | 31 | 31 | 31 | 31 | 31 | 31 | 31 |
|  | Fast spindle density | Correlation | .300 | -.004 | .085 | .057 | .214 | .423 | -.037 | .300 | .030 | .232 | .126 | .009 |
|  |  | Significance (2-tailed) | .090 | .982 | .639 | .755 | .233 | .014 | .838 | .090 | .870 | .195 | .484 | .961 |
|  |  | df | 31 | 31 | 31 | 31 | 31 | 31 | 31 | 31 | 31 | 31 | 31 | 31 |
|  | Fast spindle frequency | Correlation | -.120 | .008 | -.028 | .079 | -.143 | -.195 | .000 | -.296 | .016 | -.208 | .209 | .030 |
|  |  | Significance (2-tailed) | .508 | .965 | .879 | .663 | .427 | .276 | .998 | .095 | .930 | .245 | .243 | .868 |
|  |  | df | 31 | 31 | 31 | 31 | 31 | 31 | 31 | 31 | 31 | 31 | 31 | 31 |
|  | Fast spindle amplitude | Correlation | .117 | .032 | -.118 | .164 | .309 | .072 | .014 | -.013 | .049 | -.259 | .197 | -.200 |
|  |  | Significance (2-tailed) | .518 | .858 | .514 | .362 | .081 | .692 | .939 | .942 | .785 | .146 | .273 | .266 |
|  |  | df | 31 | 31 | 31 | 31 | 31 | 31 | 31 | 31 | 31 | 31 | 31 | 31 |
|  | Fast spindle duration | Correlation | .167 | .049 | -.001 | -.075 | .127 | .202 | -.001 | .133 | .097 | .243 | .028 | -.196 |
|  |  | Significance (2-tailed) | .352 | .785 | .997 | .679 | .481 | .259 | .994 | .461 | .589 | .174 | .877 | .274 |
|  |  | df | 31 | 31 | 31 | 31 | 31 | 31 | 31 | 31 | 31 | 31 | 31 | 31 |
|  | Slow spindle density | Correlation | .191 | .173 | -.034 | -.157 | .219 | .209 | .089 | .074 | .248 | .181 | .012 | -.286 |
|  |  | Significance (2-tailed) | .287 | .336 | .851 | .382 | .222 | .243 | .623 | .684 | .165 | .314 | .945 | .106 |
|  |  | df | 31 | 31 | 31 | 31 | 31 | 31 | 31 | 31 | 31 | 31 | 31 | 31 |
|  | Slow spindle frequency | Correlation | .051 | .134 | -.190 | -.271 | -.086 | .126 | .161 | .159 | .099 | .190 | -.140 | -.023 |
|  |  | Significance (2-tailed) | .777 | .457 | .290 | .127 | .635 | .485 | .370 | .378 | .585 | .291 | .437 | .898 |
|  |  | df | 31 | 31 | 31 | 31 | 31 | 31 | 31 | 31 | 31 | 31 | 31 | 31 |
|  | Slow spindle amplitude | Correlation | .177 | .112 | -.108 | .128 | .344 | .137 | .088 | -.009 | .130 | -.232 | .222 | -.218 |
|  |  | Significance (2-tailed) | .325 | .535 | .550 | .478 | .050 | .447 | .628 | .960 | .470 | .194 | .214 | .223 |
|  |  | df | 31 | 31 | 31 | 31 | 31 | 31 | 31 | 31 | 31 | 31 | 31 | 31 |
|  | Slow spindle duration | Correlation | -.021 | .087 | -.159 | -.263 | -.038 | .051 | .015 | -.001 | .155 | .130 | -.133 | -.270 |
|  |  | Significance (2-tailed) | .908 | .630 | .377 | .138 | .835 | .778 | .936 | .994 | .389 | .472 | .460 | .128 |
|  |  | df | 31 | 31 | 31 | 31 | 31 | 31 | 31 | 31 | 31 | 31 | 31 | 31 |

*Note.* All cognitive task scores are z-scores; FAS – FAS letter fluency test; PSQI – Pittsburgh Sleep Quality Index; TOPF – Test of Premorbid Functioning.

**Supplement 8: Scatterplots for all correlations**

*Note.* FAS – FAS letter fluency test; PSQI – Pittsburgh Sleep Quality Index; TOPF – Test of Premorbid Functioning.

**Supplement 9: Pearson correlation coefficients controlled for age including bootstrapped 95% confidence intervals**

|  | | **Fast spindle density** | | **Slow spindle density** | **PSQI** |
| --- | --- | --- | --- | --- | --- |
|  | **Memory composite score** |  | -.004 [-.353, .499] | .173 [-.159, .539] | -.311 [-.578, .021] |
|  | **Global cognition composite score** |  | .300 [-.130, .580] | .191 [-.184, .494] | -.097 [-.381, .266] |
|  | **PSQI** |  | .009 [-.538, .395] | -.286 [-.611, .124] |  |
|  | **TOPF IQ** |  | .085 [-.340., .375] |  |  |
|  | **Digit-symbol coding test** |  | .057 [-.391, .403] |  |  |
|  | **Trail Making test A** |  | .214 [-.143, .443] |  |  |
|  | **Digit span** |  | **.423* [-.167, .722]** |  |  |
|  | **FAS** |  | .300 [-.286, .640] |  |  |
|  | **Hotel test** |  | .232 [-.047, .437] |  |  |
|  | **Trail making test B** |  | .126 [-.406, .532] |  |  |

*Note.* All correlations were controlled for age; all cognitive task scores are z-scores with higher values corresponding to better performance; spindle density was measured as spindles per minute; * indicates *p*<.05, ** indicates *p*<.01; 2-tailed correlation was calculated except for primary outcome (1-tailed, memory composite score and fast spindle density); FAS – FAS letter fluency test; PSQI – Pittsburgh Sleep Quality Index; TOPF – Test of Premorbid Functioning.

**Supplement 10: Multiple linear regression models**

| **Outcome variable** | **Predictor variable** | **Unstand.B** | **Coefficients Std. Error** | **Stand. Coefficients Beta** | **p-value** | **95% CI Lower Bound** | **95% CI Upper Bound** |
| --- | --- | --- | --- | --- | --- | --- | --- |
| Memory composite score | Fast spindle density | -.058 | .363 | -.029 | .874 | -.801 | .684 |
|  | Age | -.027 | .015 | -.319 | .084 | -.059 | .004 |
|  | Gender | .110 | .383 | .055 | .775 | -.673 | .894 |
|  | TOPF IQ | .017 | .021 | .147 | .428 | -.026 | .060 |
| Global cognition score | FSD | .263 | .172 | .232 | .138 | -.089 | .615 |
|  | Age | -.003 | .007 | -.054 | .717 | -.018 | .012 |
|  | Gender | .098 | .182 | .086 | .594 | -.273 | .469 |
|  | TOPF IQ | **.036** | **.010** | **.559** | **.001** | **.016** | **.056** |
| PSQI | Fast spindle density | -.123 | 1.292 | -.018 | .925 | -2.766 | 2.519 |
|  | Age | .085 | .055 | .281 | .129 | -.026 | .197 |
|  | Gender | .587 | 1.363 | .083 | .670 | -2.201 | 3.374 |
|  | TOPF IQ | .030 | .074 | .075 | .688 | -.122 | .182 |
| Digit-symbol coding test | Fast spindle density | -.101 | .364 | -.049 | .783 | -.846 | .643 |
|  | Age | -.003 | .015 | -.036 | .839 | -.035 | .028 |
|  | Gender | .674 | .384 | .327 | .090 | -.111 | 1.460 |
|  | TOPF IQ | .040 | .021 | .338 | .068 | -.003 | .082 |
| Trail Making Test A (reversed) | Fast spindle density | .316 | .332 | .155 | .349 | -.364 | .996 |
|  | Age | .025 | .014 | .287 | .081 | -.003 | .054 |
|  | Gender | .119 | .351 | .058 | .737 | -.598 | .836 |
|  | TOPF IQ | **.048** | **.019** | **.413** | **.017*** | **.009** | **.088** |
| Digit span | Fast spindle density | **.857** | **.329** | **.419** | **.014*** | **.184** | **1.531** |
|  | Age | .011 | .014 | .121 | .450 | -.018 | .039 |
|  | Gender | -.214 | .348 | -.104 | .543 | -.925 | .497 |
|  | TOPF IQ | .036 | .019 | .305 | .069 | -.003 | .075 |
| FAS | Fast spindle density | .512 | .328 | .250 | .129 | -.158 | 1.182 |
|  | Age | .010 | .014 | .115 | .468 | -.018 | .039 |
|  | Gender | .066 | .346 | .032 | .849 | -.640 | .773 |
|  | TOPF IQ | **.055** | **.019** | **.468** | **.007**** | **.016** | **.093** |
| Hotel test (reversed) | Fast spindle density | .526 | .373 | .257 | .169 | -.237 | 1.290 |
|  | Age | -.006 | .016 | -.072 | .691 | -.039 | .026 |
|  | Gender | -.327 | .394 | -.159 | .413 | -1.133 | .478 |
|  | TOPF IQ | .015 | .021 | .126 | .499 | -.029 | .059 |
| Trail Making Test B (reversed) | Fast spindle density | .108 | .336 | .053 | .750 | -.580 | .796 |
|  | Age | -.003 | .014 | -.036 | .823 | -.032 | .026 |
|  | Gender | .245 | .355 | .119 | .496 | -.481 | .971 |
|  | TOPF IQ | **.063** | **.019** | **.535** | **.003**** | **.023** | **.102** |
| Memory composite score | Slow spindle density | .109 | .110 | .171 | .332 | -.117 | .334 |
|  | Age | -.025 | .015 | -.296 | .104 | -.057 | .006 |
|  | Gender | .098 | .363 | .049 | .789 | -.644 | .840 |
|  | TOPF IQ | .017 | .020 | .149 | .410 | -.025 | .059 |
| PSQI | Slow spindle density | -.611 | .382 | -.273 | .120 | -1.391 | .170 |
|  | Age | .075 | .053 | .249 | .162 | -.032 | .183 |
|  | Gender | .527 | 1.258 | .075 | .678 | -2.045 | 3.099 |
|  | TOPF IQ | .025 | .070 | .062 | .725 | -.119 | .169 |
| Memory composite score | PSQI | -.092 | .049 | -.325 | .072 | -.193 | .009 |
|  | Age | -.019 | .015 | -.226 | .208 | -.050 | .011 |
|  | Gender | .144 | .350 | .072 | .682 | -.570 | .859 |
|  | TOPF IQ | .019 | .020 | .166 | .340 | -.021 | .059 |

*Note.* All cognitive task scores are z-scores with higher values corresponding to better performance; spindle density was measured as spindles per minute; gender was coded as 0=male and 1=female; * indicates *p*<.05, ** indicates *p*<.01; FAS – FAS letter fluency test; PSQI – Pittsburgh Sleep Quality Index; TOPF – Test of Premorbid Functioning.

**Supplement 11: Pearson correlation coefficients controlled for age by lithium intake including bootstrapped 95% confidence intervals**

|  | | **Fast spindle density** | | **Slow spindle density** | | **PSQI** | |
| --- | --- | --- | --- | --- | --- | --- | --- |
| *Lithium* | | *no* | *yes* | *no* | *yes* | *no* | *yes* |
|  | **Memory composite score** | -.223  [-.565, .490] | .509  [.000, .912] | .056  [-.385, 569] | .409  [-.165, .844] | **-.529***  **[-.774, -.154]** | .172  [-.474, .778] |
|  | **Global cognition composite score** | **.441***  **[-.016, .672]** | -.056  [-.697, .506] | .429  [-.004, .788] | -.250  [-.796, .211] | -.263  [-.571, .290] | .203  [-.354, .725] |
|  | **PSQI** | -.041  [-.767, .381] | .263  [-.592, .917] | -.352  [-.767, .167] | -.030  [-.772, .644] |  |  |
|  | **TOPF IQ** | .242  [-.253, 538] | -.256  [-.710, .515] |  |  |  |  |
|  | **Digit-symbol coding test** | .378  [.038, .668] | -.584  [-.903, -.174] |  |  |  |  |
|  | **Trail Making test A** | .310  [-.082, .558] | -.062  [-.661, .568] |  |  |  |  |
|  | **Digit span** | **.671****  **[-.118, .897]** | -.075  [-.501, .372] |  |  |  |  |
|  | **FAS** | **.548***  **[-.198, .836]** | -.370  [-.827, .107] |  |  |  |  |
|  | **Hotel test** | .314  [-.055, .618] | .090  [-.536, .625] |  |  |  |  |
|  | **Trail making test B** | .353  [-325, .658] | -.210  [-.867, .514] |  |  |  |  |

*Note.* Only correlations specified in the aims are reported here; all correlations were controlled for age; all cognitive task scores are z-scores with higher values corresponding to better performance; spindle density was measured as spindles per minute; * indicates *p*<.05, ** indicates *p*<.01; FAS – FAS letter fluency test; PSQI – Pittsburgh Sleep Quality Index; TOPF – Test of Premorbid Functioning.

**Supplement 12: Moderation analyses**

| **Outcome variable** | **Predictor variable** | **Unstand.B** | **Coefficients Std. Error** | **Stand. Coefficients Beta** | **p-value** | **95% CI Lower Bound** | **95% CI Upper Bound** |
| --- | --- | --- | --- | --- | --- | --- | --- |
| Global cognition composite score | Fast spindle x lithium | -.237 | .211 | -.232 | .271 | -.670 | .196 |
|  | Fast spindle density | **.266** | **.113** | **.481** | **.026*** | **.033** | **.498** |
|  | Lithium intake | -.266 | .205 | -.234 | .205 | -.687 | .154 |
|  | Age | -.001 | .009 | -.031 | .862 | -.019 | .016 |
|  | Gender | -.128 | .211 | -.113 | .547 | -.560 | .303 |
| Digit span | Fast spindle x lithium | -.664 | .336 | -.358 | .058 | -1.351 | .024 |
|  | Fast spindle density | **.694** | **.180** | **.694** | **<.001**** | **.326** | **1.063** |
|  | Lithium intake | -.486 | .326 | -.236 | .147 | -1.154 | .182 |
|  | Age | .012 | .014 | .130 | .401 | -.016 | .039 |
|  | Gender | -.442 | .335 | -.215 | .197 | -1.128 | .243 |
| FAS | Fast spindle x lithium | -.638 | .356 | -.344 | .084 | -1.367 | .092 |
|  | Fast spindle density | **.555** | **.191** | **.555** | **.007**** | **.163** | **.946** |
|  | Lithium intake | -.616 | .346 | -.299 | .086 | -1.325 | .093 |
|  | Age | .011 | .014 | .129 | .433 | -.018 | .041 |
|  | Gender | -.297 | .355 | -.144 | .411 | -1.025 | .431 |

*Note.* All cognitive task scores are z-scores with higher values corresponding to better performance; spindle density was measured as spindles per minute and standardised (*M*=0, *SD*=1); gender was coded as 0=male and 1=female; lithium was coded as 0=no lithium intake, 1=lithium intake; * indicates *p*<.05, ** indicates *p*<.01; FAS – FAS letter fluency test.
